# Appendicular Neuroendocrine Tumours: An Unusual Cause of Hydronephrosis

**DOI:** 10.7759/cureus.62774

**Published:** 2024-06-20

**Authors:** Dragos Puia, Marius Ivanuta, Catalin Pricop

**Affiliations:** 1 Urology, University for Medicine and Pharmacy "Grigore T. Popa", Iasi, ROU

**Keywords:** scintigraphy, synaptophysin, chromogranin, obstructive hydronephrosis, appendiceal neuroendocrine tumor

## Abstract

Although neuroendocrine tumours (NETs) are predominantly located in the gastrointestinal tract, pancreas, and lungs, they can also occur in uncommon places such as the biliary system, prostate, breast, head, neck, and even the spinal cord. We present the case of a 30-year-old woman who was referred to the urology clinic for right ureterohydronephrosis. Because the contrast-enhanced CT scan did not show signs of kidney stones or an upper urothelial tract cell carcinoma and was combined with renal scintigraphy, the kidney was not functional, and a left nephrectomy was performed. During the surgery, it was observed that the appendix was attached to the ureter by a tiny tumour. In addition, an appendectomy was also conducted. The pathological test indicated the presence of a NET that had invaded the ureter. The diagnosis was confirmed by immunohistochemical staining. The tissue has been positive for chromogranin and synaptophysin staining. Our work highlights the infrequency and difficulty of diagnosing NETs that invade the ureter. Conducting a thorough histological evaluation in patients with uncertain histopathological diagnoses is essential.

## Introduction

Neuroendocrine cells have a vital function in connecting the endocrine system. In addition, they secrete and distribute hormones that regulate the functioning of the organs in which they are located. Neuroendocrine cells in the gastrointestinal tract produce hormones that stimulate the secretion of digestive juices and regulate food movement through the intestines. Neuroendocrine tumours (NETs) are predominantly located in the gastrointestinal tract, pancreas, and lungs, making up 82% of all cases, according to Cuthbertson et al. [1]. In addition, NETs can develop in several glands, including the parathyroid, thyroid, adrenal, and pituitary glands. They can also occur in uncommon places such as the biliary system, prostate, breast, head, neck, and even the spinal cord [2]. NETs can also occur in the skin, breast, bladder, and kidneys, but these sites are considered uncommon for this kind of tumour [3]. The varied distribution of NETs across several anatomical regions emphasises the diversity of these tumours and the requirement for thorough diagnostic and therapeutic strategies customised to each particular location.

## Case presentation

A 30-year-old woman with a history of kidney stones who spontaneously passed two years prior presented to the hospital for slight right thoracic pain. She was evaluated in the emergency department. Because right hydronephrosis was highlighted at ultrasound, she was referred to the urology clinic for further investigations and treatment.

Diagnostic assessment

**Figure 1:**
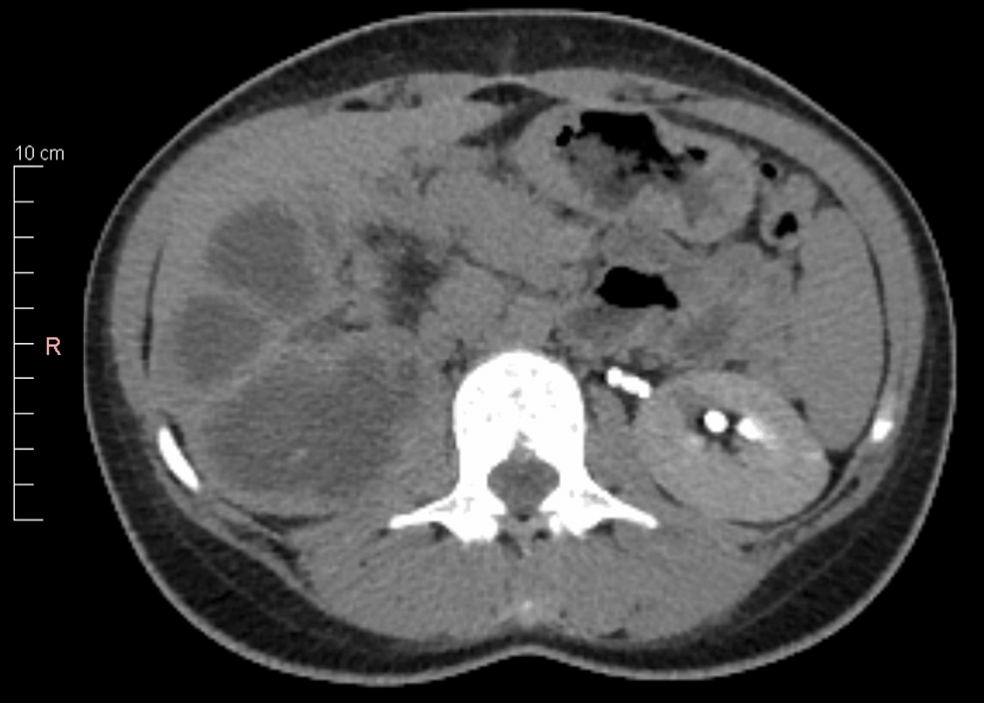
Contrast-enhanced CT image showing right hydronephrosis and non-functional kidney CT: computed tomography

At admission, most of the laboratory tests were in the normal range, as shown in Table 1. Ultrasound confirmed a right hydronephrosis with no visible renal parenchyma. The contrast-enhanced CT scan showed the right kidney as a substantial cystic mass with multiple septa, with the dilatation of the lumbar ureter suggestive of ureterohydronephrosis, and no sign of kidney stones. Based on this, the presumptive diagnosis has been an extrinsic obstruction. The kidney revealed no signs of functionality, and the parenchyma was relatively thin (Figure 1).

**Table 1:** Laboratory findings at admission

Blood test	Patient’s values	Normal range
White cell count	17x 10^9^/L	3.6-11.0 x 10^9^/L
Red cell count	3.4x 10^9^/L	3.8-5.8 x 10^9^/L
Haemoglobin	106 g/L	115-165 g/L
Glucose	82 mg/dL	70-99 mg/dL
Creatinine	0.74 mg/dL	0.8-1.3 mg/dL
Urea	1.1 mmol/L	1.2-3 mmol/L
Prothrombin time	11 seconds	10-14 seconds
Activated partial thromboplastin time	28 seconds	24-37 seconds

Therapeutic interventions

**Figure 2:**
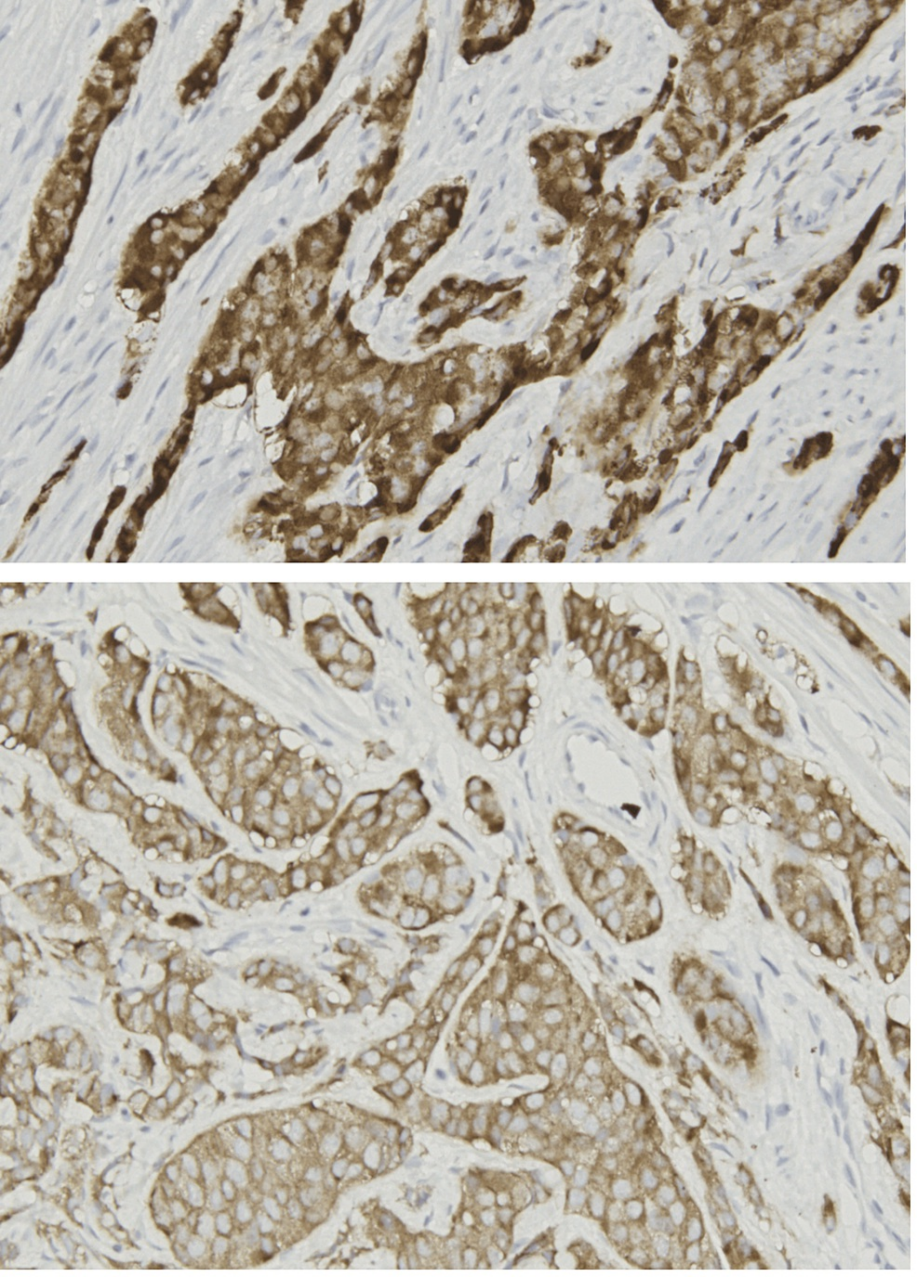
Synaptophysin and chromogranin staining (x20)

Considering imagistic and laboratory findings, the insertion of a double-J stent was attempted. The patient was given 1 g of ceftriaxone two hours before the intervention and then one dose every 12 hours until the result of the urine culture was obtained. The insertion failed because of an impassable obstacle suggestive of an extrinsic invasion. A percutaneous nephrostomy was placed, with the evacuation of sterile urine. Three weeks later, there was no drainage through the nephrostomy tube, and the renal scintigram revealed a split function of 22% in the right kidney. In this condition, a nephrectomy was performed. Intraoperatively, the appendix was adherent to the ureter through a small tumoral mass; in this situation, appendicectomy was also performed. Pathological examination suggested a NET invading the ureter, while the renal parenchyma showed tubular atrophy and chronic non-bacterial inflammation. Being an unusual type of tumour, immunohistochemical staining was performed to confirm the diagnosis. The tissue proved positive for chromogranin and synaptophysin staining (Figure 2).

Considering that NETs usually arise from the gastrointestinal tube, a colonoscopy was performed two months after surgery. Fortunately, the result was normal, and for this reason, the patient did not receive additional treatment. The follow-up did not reveal a tumour relapse 18 months after surgery.

## Discussion

NETs are relatively rare encounters. According to Albishi et al., they have an annual incidence between 2.5 and five cases per 100,000 individuals [4]. Involvement of the urinary tract is extremely rare, with less than 1% of NETs involving the genitourinary system. According to Olweny et al., primary renal carcinoids, a subtype of genitourinary NETs, comprise only 19% of reported cases worldwide [5].

Usually, NETs have some symptoms like fatigue, rectal bleeding, unexplained weight loss, high blood pressure, and changes in stool size, shape, or colour. In our case, the patient had light lumbar pain that could be secondary to the kidney obstruction. Although contrast-enhanced CT scans are often used to investigate the aetiology of hydronephrosis, according to Partelli et al., this investigation has low sensitivity (26%) in detecting lymph node metastases in nonfunctioning pancreatic NETs compared to other methods like EUS and 68Ga-DOTATOC PET [6]. Moreover, according to Duan et al., imaging studies, particularly PET/MR imaging, are crucial in diagnosing NETs by characterising the primary tumour, staging, and differentiating them from other conditions due to high soft tissue contrast [7]. This could be a reason why we did not have a preoperative diagnosis.

In our patient, histopathological evaluation with hematoxylin-eosin staining could not give a definitive diagnosis because the tissue's appearance was unusual for its location in the ureter. An immunohistochemical examination was performed. The tissue was positive for chromogranin and synaptophysin staining. Chromogranin, particularly chromogranin A and chromogranin B, has a vital function in the identification of NETs. The European Neuroendocrine Tumour Society recognises CgA as a reliable blood tumour marker for patients with NETs. According to Tsai et al., higher levels of CgA are associated with a greater number of tumours in patients with gastroenteropancreatic NET [8]. However, CgB has demonstrated importance as a possible biochemical indicator for identifying NETs in different locations, with elevated levels reported in patients compared to healthy individuals, especially in intestinal NETs. Synaptophysin is also essential for the diagnosis and prognosis of NETs. According to Hinterleitner et al., NETs may be accurately assessed for tumour stages, grading, and prognosis using this dependable biomarker [9]. Synaptophysin is selectively present in neuroendocrine tissues and has greater sensitivity than chromogranin A in the diagnosis of gastroenteropancreatic NETs, with a higher rate of positive expression [10]. In addition, there is a significant correlation between the expression of synaptophysin and higher tumour stages, the incidence of metastasis, histological grading, and tumour proliferation. This makes synaptophysin a suitable marker for monitoring the course of tumours and the response to treatment in patients with NETs [11].

Current treatment options for NETs include surgery, locoregional therapy, systemic therapy like chemotherapy, somatostatin analogues, tyrosine kinase inhibitors, mTOR inhibitors, and peptide receptor radionuclide therapy [12]. Although this was the case for our patient, these medications have demonstrated effectiveness in controlling NETs by enhancing progression-free survival and decreasing tumour size. Furthermore, there is ongoing research on immune checkpoint inhibitors, which are novel treatment alternatives. However, it is still uncertain what specific benefits they offer compared to current medications [13]. The changing treatment options for NETs highlight the significance of customised methods that take into account the specific features of the tumours in order to maximise results and minimise harmful effects. While these medications show promise in treating NETs and may improve the length of time without disease progression, none of the currently known medicinal treatments will cure the condition. For the moment, according to Yalcin et al., surgery remains the only curative approach [14].

## Conclusions

This report underscores the rarity and diagnostic challenge of NETs invading the ureter. A comprehensive histopathological examination is mandatory for patients with ambiguous histopathological diagnoses. The report further emphasises the need for long-term follow-up, given the potential for aggressive behaviour and recurrence of these tumours. Lastly, it advocates for more research to establish standardised treatment protocols for this rare entity.
